# A Single Institution's Overweight Pediatric Population and Their Associated Comorbid Conditions

**DOI:** 10.1155/2014/517694

**Published:** 2014-02-13

**Authors:** Sigrid Bairdain, Chueh Lien, Alexander P. Stoffan, Michael Troy, Donald C. Simonson, Bradley C. Linden

**Affiliations:** ^1^Department of Pediatric Surgery, Boston Children's Hospital, Harvard Medical School, Boston, MA 02115, USA; ^2^Division of Endocrinology, Brigham & Women's Hospital, Harvard Medical School, Boston, MA 02115, USA; ^3^Pediatric Surgical Associates, Children's Hospitals and Clinics of Minnesota, 2530 Chicago Avenue, South, Suite 550, Minneapolis, MN 55404, USA

## Abstract

*Background*. Obesity studies are often performed on population data. We sought to examine the incidence of obesity and its associated comorbidities in a single freestanding children's hospital. *Methods*. We performed a retrospective analysis of all visits to Boston Children's Hospital from 2000 to 2012. This was conducted to determine the incidence of obesity, morbid obesity, and associated comorbidities. Each comorbidity was modeled independently. Incidence rate ratios were calculated, as well as odds ratios. *Results*. A retrospective review of 3,185,658 person-years in nonobese, 26,404 person-years in obese, and 25,819 person-years in the morbidly obese was conducted. Annual rates of all major comorbidities were increased in all patients, as well as in our obese and morbidly obese counterparts. Incidence rate ratios (IRR) and odds ratios (OR) were also significantly increased across all conditions for both our obese and morbidly obese patients. *Conclusions*. These data illustrate the substantial increases in obesity and associated comorbid conditions. Study limitations include (1) single institution data, (2) retrospective design, and (3) administrative undercoding. Future treatment options need to address these threats to longevity and quality of life.

## 1. Introduction

The increased prevalence of overweight and obese children is not new, yet it has been viewed more recently as a public health epidemic [1–3]. According to the Centers for Disease Control and Prevention (CDC), approximately 12.5 million, or 17%, of American children are obese. More recent studies show that over 10% of 2–5-year-olds would be classified as overweight, whereas this number increases to 15% in the adolescent age group [4].

Interestingly, in 2000 the US Preventive Services Task Force (USPSTF) did not find enough evidence to recommend for or against routine screening for overweight status in either children or adolescents as a means of mitigating further health sequelae, yet now the task force is revisiting this idea as of 2010 [5]. It is known that a higher prevalence of comorbid diseases attributable to obesity is seen in both adults and children, especially modifiable cardiovascular risk factors and sequelae. In the long-standing Bogalusa Heart study, body mass indexes (BMI) performed in childhood and adolescence, as a measurement for obesity, predicted intima-media thickness in adults [6].

Obesity is not only related to modifiable cardiovascular risk factors, but also to several other health-related conditions that may persist or worsen in adulthood [7, 8]. This includes asthma, orthopedic disorders, depression and anxiety, liver abnormalities, and endocrine related issues including type 2 diabetes, hyperlipidemia, and polycystic ovarian syndrome (PCOS) [9]. Further studies may reflect resolution in these conditions with increased awareness of the association between obesity and multiple comorbid conditions. However, these studies are often performed on population-based level. Therefore, we sought to examine the incidence of the diverse sequelae of obesity and morbid obesity, as evidenced by its associated comorbidities in a single, large, free-standing, children's hospital.

## 2. Methods

### 2.1. Data Source

This study was conducted with the approval of the Institutional Review Board at Boston Children's Hospital (IRB-P00001304). No individual patient health information was collected in this study. All inpatient and outpatient visits to Boston Children's Hospital between January 1, 2000, and December 31, 2012, were included in the dataset. These visit were queried through the Informatics for Integrating Biology and the Bedside (i2b2) data warehouse platform (i2b2 v. 1.4; USA) implemented at the institution [10]. The i2b2 system allowed investigators to perform queries on an enterprise data repository through its web-based interface [11]. Selected demographics information in Epic computer system and information on hospital-billed diagnoses in Cerner, in the form of International Classification of Diseases, 9th Revision, Clinical Modification (ICD-9) codes, were available through i2b2.

The data collected was not stratified based on age, gender, nor whether the visit was an inpatient or outpatient visit in nature. Using the i2b2 web client, queries were entered in the form of inclusion or exclusion “filter” groups based on ICD-9 codes and date criteria, with date criteria based on the start date of the encounters. Our queries and calculations are described in Table 1. We obtained counts for different “patient groups” by year based on the respective administrative diagnosis. Temporal constraints were set to treat all groups independently as opposed to have the “filter” groups occur in the same financial encounter. To account for the children and adolescents in our study population, both adult and pediatric ICD-9 criteria were used in determining obesity status, utilizing both the BMI categorization standard as defined by World Health Organization and the percentile standard as defined by American Medical Association [12, 13]. ICD-9 code criteria for obesity status and comorbid conditions are further described in Table 2. A single condition was considered met if one or more of its ICD-9 criteria were coded. To help limit the number of “double-counting,” a patient was only counted once in each “patient group” regardless of how many ICD-9 criteria of the same condition the patient has met in that particular year.

### 2.2. Statistical and Data Analysis

The main comorbid conditions included diabetes mellitus, hypertension, hyperlipidemia, sleep apnea, degenerative joint disease, asthma, and depression. Overall rates of these related comorbid conditions were calculated for obese and morbidly obese versus nonobese patients, as well as the annual increase for all patients over that same time period. Other obesity-related comorbid conditions that we examined included acanthosis nigricans, nonalcoholic steatohepatitis (NASH), PCOS, glucose intolerance, insulin resistance, left ventricular hypertrophy (LVH), elevated blood pressure, and pseudotumor cerebri. The incidence rate ratio (IRR) was then calculated to see if obese and morbidly obese patients were disproportionately affected by these conditions. An odds ratio (OR) was also calculated.

## 3. Results

**Figure 1:**
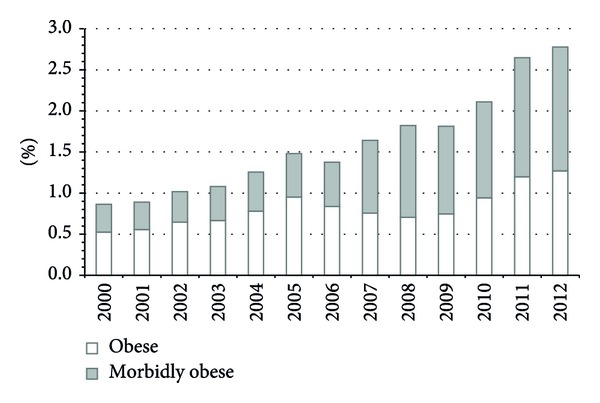
This figure illustrates the overall percentage (%) of patients coded as obese and morbidly obese from 2000–2012.

**Figure 2:**
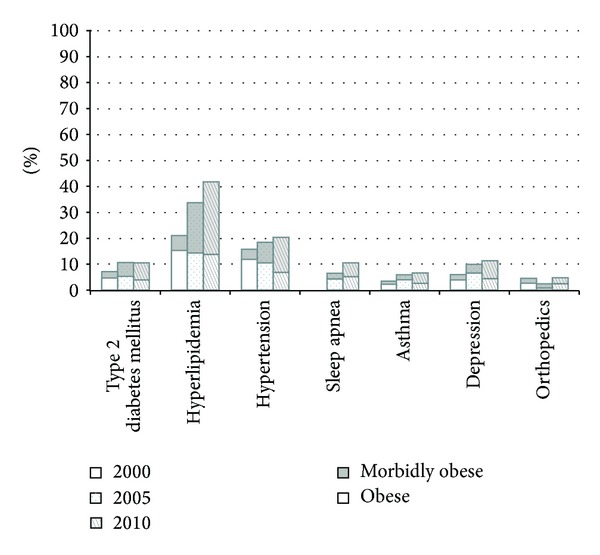
This figure illustrates the percentage (%) of patients with major comorbid conditions coded as obese and morbidly obese (not obese) at time points 2000, 2005, and 2010.

**Figure 3:**
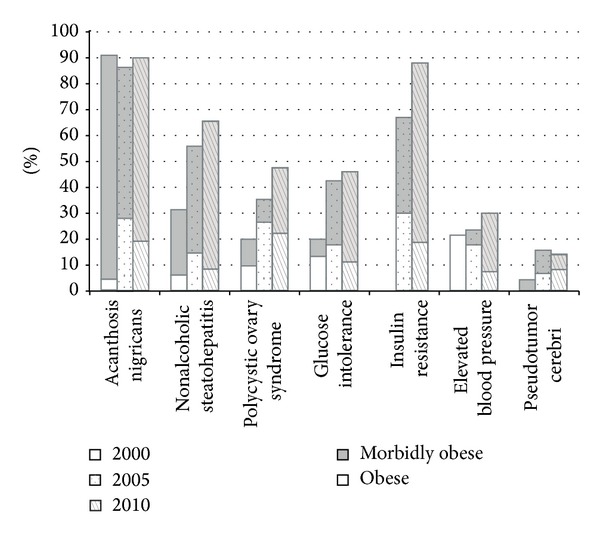
This figure illustrates the percentage (%) of patients with “preconditions” coded as obese and morbidly obese (not obese) at time points 2000, 2005, and 2010.

In total, a retrospective review of 3,185,658 person-years in nonobese, 26,404 person-years in obese, and 25,819 person-years in the morbidly obese children was conducted. The annual rate of patients coded with obesity increased from 0.86% in 2000 to approximately 2.78% in 2012 at our institution as shown in Figure 1. Figure 1 also illustrates that the annual rate of patients coded with morbid obesity increased from 0.34% in 2000 to 1.51% in 2012. When comparing differing major comorbid conditions, it appeared that the proportions of patients coded as obese or morbidly obese remained constant at the various time points including 2000, 2005, and 2010. On the other hand, when comparing the differing preconditions, it appeared that there was a slight increase in the proportions of patients coded as morbidly obese versus obese. This was especially prominent in the cohort of endocrine conditions including acanthosis nigricans, PCOS, and insulin resistance. A summary of the findings was displayed in Figures 2 and 3.

The annual rate of all major comorbidities (diabetes mellitus, hypertension, hyperlipidemia, sleep apnea, degenerative joint disease, asthma, and depression) increased in all pediatric patients over the study period. Other obesity-related comorbid conditions, or preconditions, including acanthosis nigricans, NASH, PCOS, glucose intolerance, insulin resistance, LVH, elevated blood pressures, hypertension, and pseudotumor cerebri, were also increased among all pediatric patients over the study period. However, when comparing annual rates in obese patients alone, the annual rates for obese patients were higher across all categories as compared to the annual rates calculated in all patients (Table 3).

All of the obesity-related comorbidities were higher among obese versus non-obese patients over the time period from 2000 to 2012. All comorbidities were also higher in the morbidly obese versus non-obese patients over the same time period. All of the “preconditions” were noted to have the same findings; all the “preconditions” were noted to be higher among obese, as were morbidly obese patients versus non-obese patients from 2000 to 2012. The OR did approximate the IRR with respect to all these conditions. These findings are seen in Table 3.

## 4. Discussion

This current study illustrates the change in the spectrum of obesity-related comorbid conditions in an overweight, pediatric population at a single institution over the past decade utilizing a hospital database system. As expected, children continue to experience the same comorbidities and preconditions associated with obesity as compared to adults. Among the weaknesses of this study, perhaps the most glaring is that this administrative database may have underestimated the overall incidence of obesity, the overall rate of obesity being only 2-3% at our institution. However, in spite of this, the spectrum of comorbidities seen in our pediatric patients and the knowledge of these have significant clinical implications. Both the medical and surgical treatment of adult obesity has led to associated refinement of treatment modalities; therefore, we hope to do the same with these data that identify the vast host of comorbidities that are most injurious to our children.

On a national level, May et al. have recently published results regarding cardiovascular sequelae in the *National Health and Nutrition Examination Survey (NHANES)* from 1999 to 2008 [14]. Again, the major cardiovascular risk factors including diabetes, hypertension, and hyperlipidemia were elevated across all cohorts but specifically increased in the overweight and obese cohorts. Similar to their study, we also continued to see a dose-response increase in reportable comorbid conditions across both our obese and morbidly obese cohorts. Unlike their study, we did not collect individual data, but rather administrative coding of diagnoses across both inpatient and outpatient visits. Accordingly, there could have been biased introduced with the respective coding, but our hope is that this large sample ameliorated some of the associated, inherent reporting bias.

Comorbid conditions including asymptomatic cardiovascular risk factors (e.g., hypertension, hyperlipidemia) and symptomatic cardiovascular risk factor (e.g., type 2 diabetes) are also prevalent in both the obese and morbidly obese subgroups. In fact, our study shows that hyperlipidemia and hypertension have the broadest effects with both our obese and morbidly-obese populations having the highest weighted OR and IRR. These results are similar to results of a population-based sample of Quebec children and adolescents, where almost one-third of their obese population had unfavorable risk factors; this was evidenced by elevations in apolipoprotein B, high-density lipoprotein cholesterol, triglycerides, insulin, glucose, C-reactive protein, and systolic blood pressure [15]. Thus, despite increased awareness of obesity and intensive medical interventions, obesity and its associated cardiovascular disease risk factors continue to represent an unabated major healthcare burden to this population as a whole.

More importantly, according to Bjørge et al., it is suggested that these comorbidities with cardiovascular implications lend to an increased rate of premature death in early adulthood after adjustment for other confounders [16]. In juxtaposition to this, Park et al. [8] report that the increased risk for morbidity and mortality may be overestimated following adjustment for adult BMI; however, one of the limitations of this study includes that it was a meta-analysis of very small studies. When examining our data more closely, it is very concerning the degree to which certain “preconditions” have arisen, including acanthosis nigricans, nonalcoholic steatohepatitis, glucose intolerance, and insulin resistance. These aforementioned conditions will eventually become life-long diseases; therefore, it is imperative to continue to reduce those comorbidities with cardiovascular implications, while at the same time providing more longitudinal analysis of obesity's long-term effects.

Obesity and morbid obesity are not only involved in modifiable cardiovascular risk factors, but are also intimately involved in the respiratory system. The evidence correlating the changes in the reactivity and the nature of the pulmonary disease seen with obesity is not new. For example, the NHANES data continues to show that excess weight has stronger association with nonatopic asthma (OR: 2.46, 95% CI: 1.21, 5.21) than atopic asthma (OR: 1.31, 95% CI: 0.70–2.57) [17]. This becomes a vicious cycle for these children as they are often unable to perform activities of daily living and are less likely to perform in exercise programs, which continues to compound weight-related issues. Our data shows that, even beyond an expected increase in asthma, there has also been a substantial increase in those documented with sleep apnea. For example, our data suggests that the odds of having sleep apnea was 6.48 (95% CI: 6.12, 6.85) times more likely if a patient was coded as obese and 7.60 (95% CI: 7.07, 8.17) times more likely if a patient was coded as morbidly obese as compared to normal weight counterparts.

Similar to cardiovascular risk factors, asthma, sleep apnea, and orthopedic problems are also increased in our obese and morbidly obese cohorts. A common theme among these conditions is that they limit the child or adolescent's ability to participate in social activities, which can further alienate this ever-growing proportion of our society. For example, in adults, increasing BMI and subsequent obesity lend itself to knee pain, disability, loss of work productivity, and diminished quality of life [18]. More recent studies in children suggest that obese children report more frequent and severe joint pain with subsequent loss of function [18–21]. Our study did also evaluate the trend between obesity and morbid obesity and depression. It is well known that obesity in children and adolescents results in decreased/reduced health-related quality of life [22]. As referenced by the above orthopedic studies, depression might have been masked as complaints of increased joint pain or inability to perform activities performed by non-obese peers. Further studies are needed to elucidate this connection.

Even though our study did not examine health-related costs specifically, according to one study, the actual cost of obesity is related to the direct costs of its associated medical conditions [23]. Continued attention and US dollars have been driven toward using policy and environmental changes to help combat the obesity epidemic; only in the last decade or so had there been a shift towards medical and operative interventions. Whereas it has been shown in adults that patients with reduced comorbid conditions attained through weight-loss surgery live longer, the same conclusions have yet to be applied to the pediatric population [24]. In fact, Oyetunji et al. remarked that despite this increased prevalence of obesity and morbid obesity among children and adolescents, only approximately 2% of morbidly obese children with a major comorbidity underwent a bariatric procedure [25]. This may be due in part to a myriad of factors including necessity for higher volume centers for pediatric bariatric procedures, as well as concern for permanent procedures to be performed in children and adolescents. However, there is still a need for novel and equitable approaches to pediatric obesity.

This study had important strengths and weaknesses. In the future, we hope to have same functional database as, for example, the National Surgical Quality Improvement Program (NSQIP) database, which relies on specifically trained personnel who use well-defined terms for data collection and dissemination. We encountered visits that had no documentation regarding vital data points including height, weight, or BMI, thus contributing to the underestimation of the prevalence of obesity. Despite these aforementioned weaknesses, this study's strengths included both its size as well as the duration of the data collection. Obese patients, as well as morbidly obese patients, had significant increases in comorbid conditions including hypertension, hyperlipidemia, sleep apnea, orthopedic issues, and type 2 diabetes mellitus as evidenced by the elevated IRR, OR, and tightly controlled confidence intervals.

## 5. Conclusions

In conclusion, these data show the change in the spectrum of obesity-related comorbid conditions in this pediatric population over the past decade. As expected, the children studied have experienced the same dramatic increases in obesity as seen in the adult population and obesity was independently correlated with major comorbidities. Future research will be needed to implement other treatment options to combat the wide variety of disease sequelae.

## Figures and Tables

**Table 1:** Study design: inclusion criteria and definitions of patient groups in a specific year.

Patient group	Inclusion/exclusion filters and definitions
*All* patients	patients with encounter start dates within a specific year^a^
*All* patients *with* condition	Include (condition criteria + date criteria)
*Obese* patients	Include (obesity criteria + date criteria)
*Obese* patients *with* condition	Include (obesity criteria + date criteria)
	Include (condition criteria + date criteria)
*Obese* patients *without* condition	Include (obesity criteria + date criteria)
	Exclude (condition criteria + date criteria)
*Nonobese* patients	(*All* patients) minus (*obese* patients)
*Nonobese* patients *with* condition	(*All* patients *with* condition) minus
	(*obese* patients *with* condition)
*Nonobese* patients *without* condition	(*Nonobese* patients) minus
	(*nonobese* patients *with* condition)

^a^Data provided by the Clinical Research Informatics group at Boston Children's Hospital.

**Table 2:** Study design with ICD-9-CM filter criteria.

Condition	ICD-9-CM codes
Obesity condition	
Obese	V85.4, V85.54, 278.01, V85.3, V85.53, 278.00
Morbidly obese	V85.4, V85.54, 278.01
Major comorbid condition	
Type 2 diabetes	250.00, 250.01, 250.02, 250.03
Hyperlipidemia	272.0, 271.1, 272.2, 272.3, 272.4
Hypertension	401, 405
Sleep apnea	327.23
Asthma	493
Depression	296.2, 296.3, 300.4, 311
Orthopedic	732.2, 732.4, 736.4, 736.42
Other comorbid conditions	
Acanthosis nigricans	701.2
NASH	571.8
PCOS	256.4
Glucose intolerance	790.2
Insulin resistance	277.7
LVH	429.3
Elevated blood pressure	796.2
Pseudotumor cerebri	348.2

ICD-9-CM: International Classification of Diseases, 9th Revision, Clinical Modification; NASH: nonalcoholic steatohepatitis; PCOS: polycystic ovary syndrome; LVH: left ventricular hypertrophy.

**Table 3:** Annual increases, odds ratio (OR), and incidence rate ratios (IRR) across 2000–2012.

Comorbidity	Annual increase in all patients 2000 to 2012 (95% CI)	Annual increase in all obese patients 2000 to 2012 (95% CI)	OR in obese versus nonobese patients 2000 to 2012 (95% CI)	OR in morbidly obese versus nonobese patients 2000 to 2012 (95% CI)	IRR in obese versus nonobese patients 2000 to 2012 (95% CI)	IRR in morbidly obese versus nonobese patients 2000 to 2012 (95% CI)
Type 2 diabetes	1.078 (1.050, 1.106)	1.127 (1.003, 1.252)	7.77 (7.47, 8.08)	8.84 (8.39, 9.31)	7.91 (6.61, 9.22)	8.60 (7.15, 10.05)
Hyperlipidemia	1.092 (1.014, 1.171)	1.164 (1.043, 1.285)	38.37 (37.2, 39.5)	52.7 (50.0, 54.6)	33.70 (30.54, 36.87)	43.96 (37.47, 50.44)
Hypertension	1.084 (1.038, 1.130)	1.115 (1.000, 1.230)	14.4 (13.9, 14.9)	15.29 (15.11, 16.49)	13.78 (11.81, 15.76)	12.86 (11.37, 14.36)
Sleep apnea	1.927 (0.569, 3.285)	1.887 (0.854, 2.921)	6.48 (6.12, 6.85)	7.60 (7.07, 8.17)	4.81 (4.51, 5.10)	5.04 (4.79, 5.29)
Asthma	1.049 (1.003, 1.094)	1.130 (1.055, 1.206)	4.23 (4.12, 4.35)	4.00 (3.85, 4.16)	3.78 (3.56, 4.01)	3.23 (2.81, 3.65)
Depression	1.041 (1.003, 1.079)	1.116 (1.026, 1.207)	7.28 (7.02, 7.56)	7.15 (6.78, 7.53)	7.09 (7.75, 6.43)	6.00 (5.07, 6.93)
Orthopedic	1.059 (1.014, 1.103)	1.111 (0.898, 1.323)	2.60 (2.35, 2.88)	2.77 (2.41, 3.81)	2.81 (2.19, 3.43)	3.30 (2.45, 4.14)
Acanthosis nigricans	1.119 (1.028, 1.210)	1.120 (1.036, 1.204)	570.21 (535.49, 607)	1036 (971, 1105)	562.89 (403.52, 722.27)	1104.2 (623.23, 1585.16)
NASH	1.206 (1.057, 1.354)	1.292 (1.081, 1.503)	95 (86.83, 103.97)	160.51 (146, 176)	83.09 (64.27, 101.91)	140.19 (106.73, 173.65)
PCOS	1.079 (1.022, 1.136)	1.171 (1.084, 1.257)	40.1 (38.21, 41.99)	37.52 (35.2, 39.9)	37.73 (34.11, 41.35)	33.43 (28.56, 38.20)
Glucose intolerance	1.392 (1.106, 1.677)	1.592 (1.032, 2.152)	50.68 (47.28, 54.33)	76.63 (70.99, 82.72)	44.17 (36.36, 51.98)	54.11 (40.39, 67.84)
Insulin resistance	2.542 (0.793, 4.291)	3.263 (0.763, 5.7)	528 (472, 590)	955 (853, 969)	334.65 (214.86, 454.43)	558.57 (317.37, 799.76)
LVH	1.199 (1.067, 1.330)	1.384 (0.953, 1.815)	0.97 (0.86, 1.08)	0.65 (0.54, 0.79)	0.81 (0.58, 1.03)	0.69 (0.44, 0.94)
Elevated BP	1.369 (1.007, 1.731)	1.470 (1.077, 1.864)	28.04 (26, 30)	34.54 (31.5, 37.8)	22.68 (18.46, 26.91)	16.89 (9.96, 23.82)
Pseudotumor cerebri	1.164 (1.058, 1.270)	1.662 (0.756, 2.568)	11.28 (9.52, 13.37)	11.34 (8.9, 14.29)	10.83 (7.91, 13.74)	13.27 (5.70, 20.84)

## Abbreviations

BMI: Body mass index
IRR: Incidence rate ratio
NHANES: National Health and Nutrition Examination Survey
NSQIP: National Surgical Quality Improvement Program
OR: Odds Ratio
WLS: Weight loss surgery.
